# Local immunization program for susceptible-infected-recovered network epidemic model

**DOI:** 10.1063/1.4941670

**Published:** 2016-02-11

**Authors:** Qingchu Wu, Yijun Lou

**Affiliations:** 1 College of Mathematics and Information Science, Jiangxi Normal University, Nanchang, Jiangxi 330022, People's Republic of China; 2 Department of Applied Mathematics, The Hong Kong Polytechnic University, Hung Hom, Kowloon, Hong Kong

## Abstract

The immunization strategies through contact tracing on the susceptible-infected-recovered framework in social networks are modelled to evaluate the cost-effectiveness of information-based vaccination programs with particular focus on the scenario where individuals belonging to a specific set can get vaccinated due to the vaccine shortages and other economic or humanity constraints. By using the block heterogeneous mean-field approach, a series of discrete-time dynamical models is formulated and the condition for epidemic outbreaks can be established which is shown to be not only dependent on the network structure but also closely related to the immunization control parameters. Results show that increasing the immunization strength can effectively raise the epidemic threshold, which is different from the predictions obtained through the susceptible-infected-susceptible network framework, where epidemic threshold is independent of the vaccination strength. Furthermore, a significant decrease of vaccine use to control the infectious disease is observed for the local vaccination strategy, which shows the promising applications of the local immunization programs to disease control while calls for accurate local information during the process of disease outbreak.

Researchers have performed many studies about the dynamic immunization schemes on networks, which provide new insights into optimal strategies to effectively control the diseases. However, previous work mainly focused on the immunization over the whole population under the assumption that the vaccines supply is sufficient to cover the most of individuals at risk. In some special scenarios, especially for emerging diseases, the vaccine supply is small compared with the large amount of population size at risk. Therefore, it is pivotal to investigate a local immunization program that is confined to a specified subpopulation. In the current manuscript, the immunization strategies through contact tracing on the susceptible-infected-recovered framework in social networks are proposed. Using dynamic analysis and numerical simulations, the epidemic thresholds are found which are shown to be closely associated with the adjustable parameters for the vaccination programs. This result is different from the case for susceptible-infected-susceptible model framework where epidemic threshold is independent of the vaccination strength. Furthermore, a significant decrease of vaccine use to control the infectious disease, through comparison of static and dynamic immunization schemes, is observed for the local vaccination strategy. These results provide novel designs for disease control using immunization programs.

## I. INTRODUCTION

The immunization can be regarded as a response to the seriousness of epidemic spreading through voluntary vaccination^1^ or interventions.^2^ Novel insights into immunization programs can be gained from the study of complex networks^3,4^ from two main perspectives^5^ among others:

(i) The static immunization which is implemented before the epidemic spreading.^6^ Generally speaking, there are two basic schemes following this idea, the random immunization and the targeted immunization,^3^ and other multiple variant strategies, such as the acquaintance immunization^7^ and inverse targeting immunization,^6^ with applications to time-varying networks^8^ and multiplex networks.^9^
(ii) The dynamic immunization where the program is implemented during the epidemic outbreak. Since an individual's vaccination decision is made mainly based on the epidemic seriousness while sometimes, the novel effective and safe vaccine can only be developed and mass-produced after the emergence of infectious diseases triggered by new pathogens, the dynamic immunization is always implemented in realistic situations in most cases.^1,10^

Similar to individual's behavioral responses to infectious diseases,^11,12^ the dynamic immunization can be well-adjusted based on transmission information during the epidemic spreading. As soon as an infectious disease begins to spread in the population, an effective immunization program should be initiated which may be adjusted according to the disease prevalence and the program terminates when the disease dies out. Therefore, the epidemic information-based immunization allows us to take the advantage of the interplay between the immunization response and the epidemic spreading. Motivated by this idea, researchers have evaluated the efficacy of various dynamic immunization schemes by using network models with direct immunization^13–15^ or other modelling approaches. For example, Shaban *et al*.^16^ formulated real-time susceptible-infected-recovered (SIR) vaccination models for contact tracing in a network with a specific degree distribution by a branching process approximation. Nian and Wang^17^ proposed a strategy to immunize the neighbors of an infected node. Ruan *et al*.^10^ studied an information-driven vaccination program and found that strengthening the information diffusion can reduce the final vaccination fraction. Wang *et al*.^18^ investigated the interplay between information spreading and disease dynamics in an information-driven vaccination program. Jo and Baek^19^ and Fu *et al*.^20^ evaluated efficiencies of immunization schemes in the susceptible-infected-recovered-susceptible (SIRS) and susceptible-infected-susceptible (SIS) networks, respectively. Zhang *et al*.^21^ investigated the impact of subsidy policies on vaccination decisions under the voluntary vaccination. More recently, Takaguchi *et al*.^22^ proposed an immunization strategy based on observer placement, which is shown to be very efficient for disease control in the clustering networks.

Although providing novel insights into the cost-effectiveness of various immunization approaches, previous studies mainly focused on the immunization over the whole population (denoted by *W* in this manuscript). However, in reality, the vaccine supply is limited compared with the large population size at risk, especially for those diseases triggered by novel pathogens. Furthermore, vaccines may deliver to only a partial population due to other economic or humanity constraints. Therefore, it becomes much more realistic to consider a local immunization program that only covers a specified subpopulation (denoted by Ω in what follows).^23^ It becomes of interest to evaluate the cost-effectiveness of static as well as dynamic immunization schemes for this realistic situation. A recent paper^23^ investigated the SIS epidemic model with local immunization program and showed that the condition of epidemic outbreak is not related to the immunization strength. The SIS modelling framework is well-accepted for describing infections, such as rotavirus and gonorrhea, which do not confer long-lasting immunity. However, a SIR framework is more suitable for infections such as measles, mumps, and chickenpox where individuals recover and confer lifelong immunity, which is the fundamental framework we will extend in the current paper with network structures. Furthermore, we are also interested in the comparison of predictions obtained from the immunization programs for these two network model frameworks.

In the current manuscript, we are going to propose two kinds of hypothetical immunization models, for local and global immunization programs, applicable to the SIR epidemic networks. Rigorous and numerical analysis will illustrate the disease transmission conditions, and the results will further be compared with previous studies on vaccination programs on the SIS framework (see the Appendix and Ref. 23). The rest of this paper is organized as follows: In Section II, we first formulate a susceptible-vaccinated-infected-recovered (SVIR) model with a global immunization program; then in Section III, we further investigate a local immunization program by theoretical analysis and simulations; finally, discussions are presented in Section IV.

## II. THE GLOBAL IMMUNIZATION PROGRAM

In this section, we extend the SIR epidemic model to SVIR models with the consideration of a global immunization scheme. Herein, the *V* state represents the immunized population through vaccination. Intuitively, one possible efficient immunization strategy is to directly immunize all the susceptible nodes connected to infected nodes, refereed to as the high-risk nodes^17^ which are likely to be infected by their infectious neighbors in the following infection wave. These high-risk nodes can be found through contact tracing theoretically.^16,24,25^ However, in practice, it is not easy to locate all these high-risk nodes and there exists a discount rate. Hence, we introduce an adjustable parameter *δ*, denoted as the tracing rate or immunization rate, to account the efficiency of tracing and immunizing these high-risk nodes. Suppose a susceptible node will get infected by one infectious neighbor with rate *β*. Then a susceptible node with *s* infected neighbors changes its state with the following probabilities:^19^

$$\begin{array}{c} P(S\to S)={\left(1-\delta \right)}^{s}{\left(1-\beta \right)}^{s},\\ P(S\to V)=1-{\left(1-\delta \right)}^{s}:=w_{1}(s),\\ P(S\to I)={\left(1-\delta \right)}^{s}[1-{\left(1-\beta \right)}^{s}]:=w_{2}(s). \end{array}$$ (1)

Following Ref. 26, we assume the network is randomly generated according to the degree distribution $P(k)∼k^{-\alpha }$ with *α* ∈ (2, 3] as many real networks to incorporate the heterogeneity of individuals. This assumption implies that the connectivity of nodes is uncorrelated. We denote $S_{k}(t),\;V_{k}(t),\;I_{k}(t),\;R_{k}(t)$ as the relative densities of susceptible, vaccinated, infected, and recovered nodes in the population with degree *k* at time step *t*, respectively, with *k* = *k*_0_, $k_{0}+1,\;\cdots ,$ *k_c_*, where *k*_0_ and *k_c_* are the minimal and maximal degrees. Assuming *I_k_*(0) ≃ 0 and *R_k_*(0) = 0 for each *k*, then the probability Θ(*t*) of a randomly selected node connecting to an infected individual can be formulated as^27,28^

$$\Theta (t)≃\frac{\sum_{k}\left(k-1)P(k)I_{k}(t\right)}{\sum_{k}kP(k)}=\frac{\sum_{k}\left(k-1)P(k)I_{k}(t\right)}{\left\langle k\right\rangle }.$$

Then the probability that a node of degree *k* has exactly *s* infected neighbors is given by the binomial distribution^29^

$$\text{Bin}(k,s)=\left(\begin{array}{c} k\\ s \end{array}\right)\Theta^{s}{\left(1-\Theta \right)}^{k-s}.$$

Taking the expectation of the stochastic variable *w*_1_(*s*) with respect to the above defined binomial distribution gives the probability with which a susceptible node of degree *k* is vaccinated

$$E[w_{1}(s)]=1-\sum_{s}\text{Bin}(k,s){\left(1-\delta \right)}^{s}=1-{\left(1-\delta \Theta \right)}^{k}.$$

Similarly, a susceptible node of degree *k* gets infected with probability

$$\begin{array}{c} E[w_{2}(s)]=\sum_{s}\text{Bin}(k,s){\left(1-\delta \right)}^{s}[1-{\left(1-\beta \right)}^{s}]\\ ={\left(1-\delta \Theta \right)}^{k}-{\left[1-(\delta +\beta -\delta \beta )\Theta \right]}^{k}. \end{array}$$

In the present paper, we employ the widely used discrete-time approach,^29–31^ capable of accounting the periodicity feature in daily life or day-night changes,^8^ in the above process of state changes during disease transmission. If we assume that an infected node recovers and simultaneously achieves the perpetual immunization to the pathogen with rate *γ*, then the discrete-time epidemic process can be described in a mean-field form

$$\begin{array}{c} \;S_{k}(t+1)=S_{k}(t)-S_{k}(t)\{E[w_{1}(s)]+E[w_{2}(s)]\},\\ \;V_{k}(t+1)=V_{k}(t)+S_{k}(t)E[w_{1}(s)],\\ \;I_{k}(t+1)=(1-\gamma )I_{k}(t)+S_{k}(t)E[w_{2}(s)],\\ \;R_{k}(t+1)=R_{k}(t)+\gamma I_{k}(t). \end{array}$$ (2)

Clearly, variables $S_{k}(t),\;V_{k}(t),\;I_{k}(t),\;R_{k}(t)$ are nonnegative and satisfy $S_{k}(t)+V_{k}(t)+I_{k}(t)+R_{k}(t)=1$ for each *k* and *t*, which can be shown from the system (2), or by their definitions.

Under the initial conditions *I_k_*(0) ≃ 0 and *R_k_*(0) = 0 for each *k*, the occurrence of an epidemic outbreak depends on the stability of the disease free equilibrium of the network model.^32^ Hence, we consider the system near the zero solution (*I_k_*(*t*) = 0 for each *k*). Then, $E[w_{2}(s)]≃\beta (1-\delta )k\Theta$ and the evolution of the infected class^33^ can be given by the linearized model of (2)

$$I_{k}(t+1)=(1-\gamma )I_{k}(t)+\beta (1-\delta )k\Theta .$$ (3)

By analyzing the Jacobian matrix of Eqs. (3), one can find that the system has a unique eigenvalue of maximum modulus, i.e., $\beta (1-\delta ){\left\langle k\right\rangle }^{-1}\sum_{k}k(k-1)P(k)+1-\gamma$, from which the epidemic threshold can be derived

$$\tau_{c}=\frac{1}{1-\delta }\frac{\left\langle k\right\rangle }{\left\langle k^{2}\rangle -\langle k\right\rangle },$$ (4)

with $\langle k^{2}\rangle =\sum_{k}k^{2}P(k)$. Here, we use the rescaled infection rate *τ* = *β*/*γ*. Then the epidemic threshold *τ_c_* determines the epidemic outbreak: if *τ* < *τ_c_*, the total infection density $I(t)=\sum_{k}I_{k}(t)P(k)$ decreases to zero (no epidemic), otherwise, *I*(*t*) first increases to a maximum and then decreases to zero (an epidemic).^32^ When *δ* > 0, the epidemic threshold is inversely proportional to 1 − *δ* value.

It is interesting to observe that the threshold index *τ_c_* derived here equals to the epidemic threshold for the SIR model with local information-based behavioral responses.^34^ It is also worthy to remark that the same disease outbreak threshold (4) can also be obtained by the branching process theory.^35^ For example, the authors in Ref. 16 studied the vaccination through the contact tracing with a general contact time and obtained the similar result, while *τ* = *β*/(*β* + *γ*) when the contact time follows an exponential distribution in that paper.

## III. THE LOCAL IMMUNIZATION PROGRAM

**FIG. 1.**
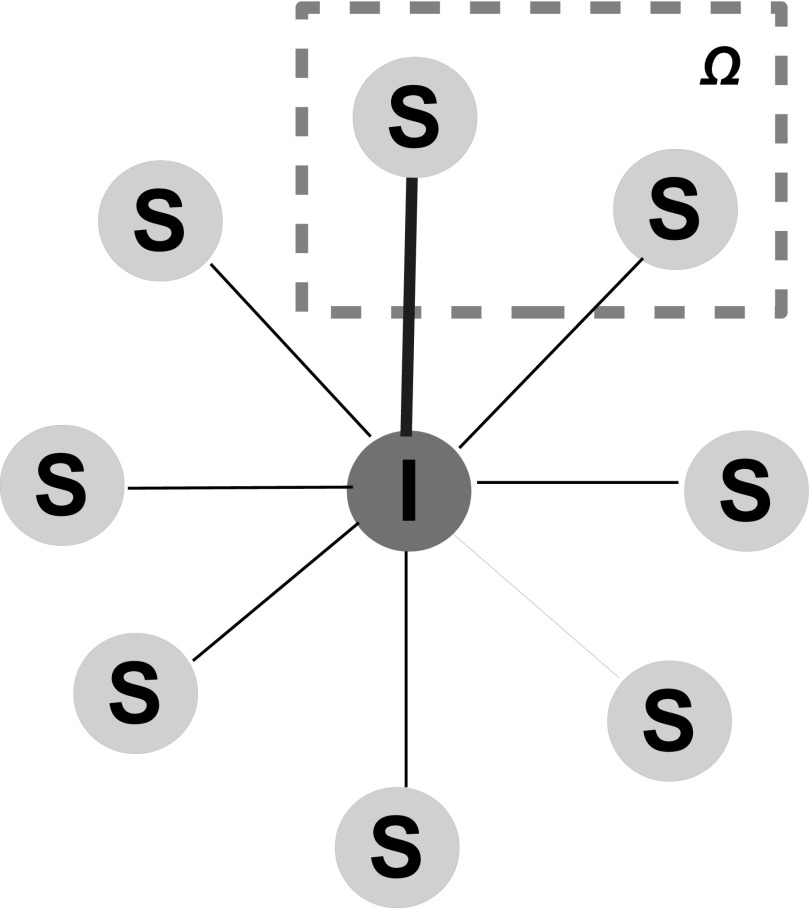
Illustrations of the contact tracing by an infected node. The central node is an infected node with 8 susceptible neighbors, among which two are in the set Ω while the others are outside Ω. Only one, out of two Ω-nodes, is traced (and also will get vaccinated) by the central node along the contact between them (indicated by a blue and thick line).

The development of optimal vaccine allocation strategies to control the epidemic spreading remains a central problem in public health and network security.^36^ Furthermore, vaccine shortages, resulting from higher-than-expected demand, interruptions in production/supply or a lack of budgets, makes it impossible to immunize almost all the nodes in the whole network and urges to design an optimal strategy minimizing the total number of vaccines or the social cost.^14^ The design of an optimal strategy in the consideration of this constraint is not only related to the high-risk nodes but also the nodes with other particular characteristics during the epidemic spreading.^6,37^ For that purpose, we introduce a local immunization program that is confined to a node set Ω, in which nodes are predefined according to special characteristics for the epidemic control, with the special reference to vaccine shortage scenario: only susceptible nodes in Ω can be vaccinated or removed while other nodes cannot. An illustrative diagram is shown in Fig. 1, where the infected node at the center is surrounded by 8 susceptible nodes and only one of two traced susceptible nodes in Ω gets vaccinated. Clearly, ∅⊂Ω⊂W with two extreme cases: when Ω=∅, the immunization scheme through vaccination is not implemented and the model reduces to a standard SIR model;^38^ while the global immunization program is implemented to cover all risky nodes for Ω = *W*. In what follows, we only consider the local immunization scenario, that is, the above inclusions are proper.

In the local immunization program, one key question is to determine which group of individuals should be traced and get immunized, in other words, to define the set Ω for the network. This question can be solved when we have no knowledge about the spreading resource^3^ by some classical static immunization strategies, such as the random immunization and targeted immunization. In order to present a comparative analysis, in this paper, we consider two kinds of Ω determined by the random immunization and targeted immunization, respectively.

When the set Ω is fixed, a subnetwork *G*_1_ formed by the nodes in Ω can be defined, and the remaining nodes together with their edges form a subnetwork *G*_2_. Since there exist links between nodes in *G*_1_ and *G*_2_, the whole network *G* can be regarded as an interdependent network^39^ where the mean-field approach is still feasible. Taking the difference between interdependent networks and a single network, we call the subnetwork *G_i_*, *i* = 1, 2, as blocks of *G* and the mean-field approach based on the blocks is correspondingly referred to as the *block heterogeneous mean-field (*HMF*) approach* (it differs from the block variable mean-field approach^40^).

### A. The random immunization case

The random immunization means that a fraction *f* of all nodes is randomly selected to be immunized,^3^ from which, the set Ω is determined. Based on the nodes in set Ω, blocks *G*_1_ and *G*_2_ can be defined accordingly. Denote $S_{k}^{\left(i\right)}(t),\;V_{k}^{\left(i\right)}(t),\;I_{k}^{\left(i\right)}(t)$, and $R_{k}^{\left(i\right)}(t)$ as the relative densities of susceptible, vaccinated, infected, and recovered nodes of *G_i_* (*i* = 1 or 2) in the population *G* with degree *k* at time step *t*, respectively. According to the discrete-time HMF approach, the dynamical model for the random immunization program is given by

$$\begin{array}{c} \;I_{k}^{\left(1\right)}(t+1)=(1-\gamma )I_{k}^{\left(1\right)}(t)+S_{k}^{\left(1\right)}(t)E[w_{2}^{\left(1\right)}(s)],\\ \;I_{k}^{\left(2\right)}(t+1)=(1-\gamma )I_{k}^{\left(2\right)}(t)+S_{k}^{\left(2\right)}(t)E[w_{2}^{\left(2\right)}(s)]. \end{array}$$

Here, the respective infection probabilities in *G*_1_ and *G*_2_ are

$$w_{2}^{\left(1\right)}(s)={\left(1-\delta \right)}^{s}[1-{\left(1-\beta \right)}^{s}],$$

and

$$w_{2}^{\left(2\right)}(s)=1-{\left(1-\beta \right)}^{s}.$$

Please note that the model does not include the dynamics of other variables for $S_{k}^{\left(i\right)}(t),\;R_{k}^{\left(i\right)}(t)$ with *i* = 1, 2 and $V_{k}^{\left(1\right)}$ which do not appear in the system describing the evolution of infectious nodes at the initial stage of disease spread.

A similar approximation analysis as in Sec. II near the disease-free equilibrium *E*_0_ gives

$$E[w_{2}^{\left(1\right)}(s)]≃\beta (1-\delta )k\Theta \;\text{and}\;E[w_{2}^{\left(2\right)}(s)]≃\beta k\Theta ,$$

with $\Theta ={\left\langle k\right\rangle }^{-1}\sum_{k}\left(k-1)P(k)[I_{k}^{\left(1\right)}+I_{k}^{\left(2\right)}\right]$.

At the early stage of an epidemic, we have

$$S_{k}^{\left(1\right)}(t)≃f\;\text{and}\;S_{k}^{\left(2\right)}(t)≃1-f$$

while

$$I_{k}^{\left(i\right)}(t)≃0,\;R_{k}^{\left(i\right)}(t)≃0\;\text{for}\;\text{each}\;i\;\text{and}\;V_{k}^{\left(1\right)}(t)≃0.$$

Let $I(t)={\left(I_{k_{0}}^{\left(1\right)},I_{k_{0}+1}^{\left(1\right)},\ldots ,I_{k_{c}}^{\left(1\right)},I_{k_{0}}^{\left(2\right)},I_{k_{0}+1}^{\left(2\right)},\ldots ,I_{k_{c}}^{\left(2\right)}\right)}^{T}$, then the local stability of *E*_0_ can be established through the following linear system for infected nodes:

$$I(t+1)=J_{1}(E_{0})I(t)$$

with

$$J_{1}(E_{0})=(1-\gamma )I_{2M\times 2M}+\beta \left(\begin{array}{cc} f(1-\delta )A & f(1-\delta )A\\ (1-f)A & (1-f)A \end{array}\right),$$

where $I_{2M\times 2M}$ is an identity matrix and *A* is a *M* × *M* positive matrix with entries $A_{kk\prime }=k(k\prime -1)P(k\prime )/\langle k\rangle$ and *M* = *k_c_* − *k*_0_ + 1.

Using the property of block matrix, one can obtain

$$\begin{array}{c} \det\left(\begin{array}{cc} f(1-\delta )A-\lambda I_{M\times M} & f(1-\delta )A\\ (1-f)A & (1-f)A-\lambda I_{M\times M} \end{array}\right)\;\\ =\det\left(\begin{array}{cc} (1-f\delta )A-\lambda I_{M\times M} & 0\\ (1-f)A & -\lambda I_{M\times M} \end{array}\right)\;\\ =\lambda^{M}\det[\lambda I_{M\times M}-(1-f\delta )A]. \end{array}$$

Using this equality, it is easy to obtain the maximal eigenvalue of *J*_1_(*E*_0_) as

$$\lambda_{\text{max}}(J_{1})=1-\gamma +\beta (1-f\delta )\lambda_{\text{max}}(A).$$

Therefore, the epidemic threshold for the random immunization case, $\tau_{c}^{r}$, is given by

$$\tau_{c}^{r}=\frac{1}{1-f\delta }\frac{\left\langle k\right\rangle }{\left\langle k^{2}\rangle -\langle k\right\rangle }.$$ (5)

It is obvious to see that the epidemic threshold is dependent of both *f* and *δ*. In addition, the epidemic threshold becomes much more sensitive to the immunization rate *δ* for larger *f* values.

### B. The targeted immunization case

The targeted immunization has been shown very effective in controlling epidemic outbreak on scale-free networks.^3^ To evaluate the efficacy of this program in this study, we choose nodes with large degrees to be vaccinated, that is, the node set Ω={v∈W:deg(v)≥K}, where deg(*v*) denotes the degree of node *v* and *K* is a control parameter. In this scenario, the whole network can be divided into two blocks:

$$G_{1}\;\text{with}\;\text{degree}\;k\geq K\;\text{and}\;G_{2}\;\text{with}\;\text{degree}\;k<K.$$

The dynamical equations for the targeted immunization case can be written as

$$\begin{array}{c} \;I_{k}^{\left(1\right)}(t+1)=(1-\gamma )I_{k}^{\left(1\right)}(t)+S_{k}^{\left(1\right)}(t)E[w_{2}^{\left(1\right)}(s)],\;k\geq K,\\ \;I_{k}^{\left(2\right)}(t+1)=(1-\gamma )I_{k}^{\left(2\right)}(t)+S_{k}^{\left(2\right)}(t)E[w_{2}^{\left(2\right)}(s)],\;k<K. \end{array}$$

Here, $E[w_{2}^{\left(1\right)}(s)]$ and $E[w_{2}^{\left(2\right)}(s)]$ are defined as before. We can obtain the linearized equations for $I(t)={\left[I_{k_{0}}^{\left(2\right)},I_{k_{0}+1}^{\left(2\right)},...,I_{K}^{\left(2\right)},I_{K+1}^{\left(1\right)},...,I_{k_{c}}^{\left(1\right)}\right]}^{T}$ for linear stability analysis of the disease free equilibrium *E*_0_

$$I(t+1)=J_{2}(E_{0})I(t).$$

The corresponding Jacobian matrix *J*_2_(*E*_0_) at *E*_0_ becomes

$$J_{2}(E_{0})=(1-\gamma )I_{M\times M}+\beta B,$$ (6)

where *B* is a *M* × *M* positive matrix with entries $B_{kk\prime }=k(k\prime -1)P(k\prime )/\langle k\rangle$ for *k* < *K* and $B_{kk\prime }=(1-\delta )k(k\prime -1)P(k\prime )/\langle k\rangle$ for *k* ≥ *K*.

It is easy to verify from (6) that the dominant eigenvalue

$$\lambda_{\text{max}}(J_{2})=1-\gamma +\beta \lambda_{\text{max}}(B),$$

with

$$\begin{array}{c} \lambda_{\text{max}}(B)=\frac{\sum_{k<K}k(k-1)P(k)}{\left\langle k\right\rangle }+(1-\delta )\frac{\sum_{k\geq K}k(k-1)P(k)}{\left\langle k\right\rangle }. \end{array}$$

Therefore, the epidemic threshold for the targeted immunization case is

$$\begin{array}{c} \tau_{c}^{t}=\frac{\left\langle k\right\rangle }{\sum_{k<K}k(k-1)P(k)+(1-\delta )\sum_{k\geq K}k(k-1)P(k)}\\ =\frac{\left\langle k\right\rangle }{\langle k^{2}\rangle -\langle k\rangle -\delta \sum_{k\geq K}k(k-1)P(k)}. \end{array}$$ (7)

It indicates that the epidemic threshold is dependent on *δ* and *K*. The epidemic threshold increases with *δ* while decreases with *K*, and the infectious disease may be controlled when *δ* is large or *K* is small enough. To compare the cost-effectiveness of the random and targeted immunization strategies, we first write Eq. (7) into the form of Eq. (5) as

$$\tau_{c}^{t}=\frac{1}{1-\tilde{f}\delta }\frac{\left\langle k\right\rangle }{\left\langle k^{2}\rangle -\langle k\right\rangle }$$ (8)

with $\tilde{f}=\frac{\sum_{k\geq K}k(k-1)P(k)}{\sum_{k}k(k-1)P(k)}$. In the targeted immunization program, we have $f=\sum_{k\geq K}P(k)$, which represents total vaccine coverage in the whole network. Next, we are going to show that $\tilde{f}>f$, which is equivalent to

$$F(K):=\sum_{k=K}^{k_{c}}\left[k(k-1)-\sum_{k}k(k-1)P(k)\right]P(k)>0.$$

Since *k*(*k* − 1) is an increasing function of *k*, there exists a threshold value *m* such that $k(k-1)\leq \sum_{j=k_{0}}^{k_{c}}j(j-1)P(j)$ when *k* ≤ *m* and $k(k-1)\geq \sum_{j=k_{0}}^{k_{c}}j(j-1)P(j)$ when *k* ≥ *m*, indicating that the function *F* increases first and then decreases across the threshold value. On the other hand, *F*(*k*_0_) = 0 and *F*(*k_c_*) > 0, and therefore, we get *F*(*K*) > 0 for all *K* > *k*_0_, which proves $\tilde{f}$ > *f*. Therefore, $\tau_{c}^{t}>\tau_{c}^{r}$ for the same *f* value with $f=\sum_{k\geq K}P(k)$ for the targeted immunization case, which implies that the epidemic threshold becomes greater for the target immunization program than that for the random immunization program with the same vaccine coverage used. Therefore, one can conclude that the targeted immunization is more efficient than the random immunization.

### C. Simulations

To verify the above theoretical analysis, we perform Monte Carlo simulations over scale-free networks generated from the standard configuration model^41^ with degree exponent *α* = 2.7. The network structure is set with size *N* = 2000, the minimal degree *k*_0_ = 3, and the maximal degree $k_{c}=\sqrt{N}≃44.72$. The recovery rate *γ* is set to be 1.0. All simulations are implemented by a parallel updating strategy in which the actual disease states of each node and its neighbors at each time step are considered. We start with a single initial infectious seed and all simulation results are obtained by taking averages of 20 random network configurations and 50 independent initial conditions for each network realization.

**FIG. 2.**
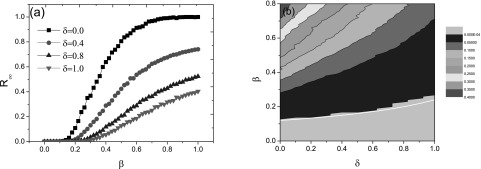
Effect of the immunization rate on the epidemic threshold and prevalence for the random immunization case when *f* = 0.5: (a) final recovered size *R_∞_* versus the infection rate *β* for different values of *δ*; (b) contour of *I*_max_ in the (*δ* – *β*) parameter plane, where the white line indicates the theoretically predicted curve determined in Eq. (5), and the light gray region corresponds to the parameter region with zero prevalence.

**FIG. 3.**
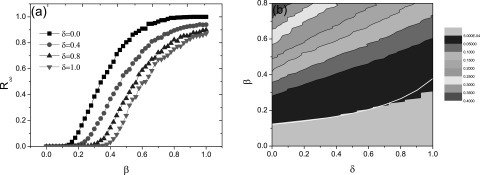
Effect of the immunization rate on the epidemic threshold and prevalence for the targeted immunization case when *K* = 10: (a) final recovered size *R_∞_* versus the infection rate *β* for different values of *δ*; (b) contour of *I*_max_ in the (*δ* – *β*) parameter plane, where the white line indicates the theoretically predicted curve determined in Eq. (7), and the light gray region to the parameter region with zero prevalence.

Fig. 2(a) illustrates the epidemic prevalence *R_∞_* (i.e., the fraction of recovered nodes at the end of the epidemic wave) as a function of infection rate *β*. This figure also shows the existence of an epidemic threshold for different immunization rates. In order to examine the validation of the theoretical results to the Monte Carlo simulation, we also consider the maximal infection density *I*_max_ for different parameters, which has been shown to be an effective index to measure the epidemic threshold of the model with infinite absorbing states.^33^ An alternative approach is based on the variability measure suggested by Shu *et al*.^42^ As illustrated in Fig. 2(b), the simulation results agree with the theoretical threshold conditions obtained in Eq. (5). Similar conclusion can be made for the targeted immunization in Fig. 3. Furthermore, increasing *δ* value can always raise the epidemic threshold *τ_c_* regardless of the random or targeted immunization case. This result is significantly different from the SVIS network model based on an SIS framework (see detailed analysis about SVIS model in the Appendix), where the parameter *δ* does not play a role for *τ_c_*.

We further investigate the impact of dynamic immunization on the final vaccine size *V_∞_* by using the immunization efficiency *Q*. When there is no infection in the network (i.e., at the steady state), we can define Ω_*X*_ as

$$\Omega_{X}=\{i\in \Omega |state\{i\}=X\},$$

where *X* is the node state, which may be *S*, *I*, *R*, or *V*. Notice that there exist infection-induced immunization nodes in Ω and therefore, Ω_*R*_ ∪ Ω_*S*_ ∪ Ω_*V*_ = Ω. Hence, the immunization efficiency for the SIR model, a function of variables *δ*, *β*, *f*, and *K*, can be expressed as

Q(δ,β,f)=Ω#SΩ#−Ω#R,

where the symbol ^*#*^Ω_*X*_ denotes the number of the elements in set Ω_*X*_.

**FIG. 4.**
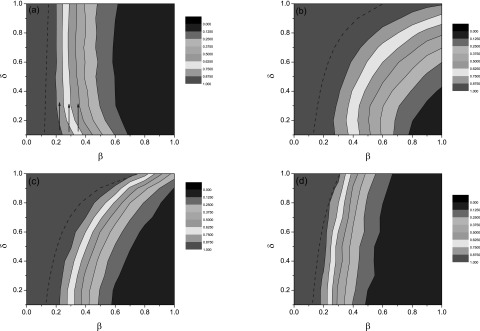
The contour plot of the immunization efficiency, where the horizontal coordinate is the infection rate *β* and the vertical coordinate is the immunization strength *δ*. Panels (a) and (b) illustrate the random immunization case for *f* = 0.2 and 0.8, respectively, while panels (c) and (d) show the targeted immunization case for *K* = 6 and 12, respectively. The dashed lines in each panel indicate the epidemic thresholds by theoretical predictions.

Fig. 4 clearly shows that the immunization efficiency index *Q* is strongly correlated with the infection rate *β* and the immunization rate *δ* for different predefined sets Ω, representing the random/targeted immunization strategies used. For each fixed *δ* value, *Q* increases as *β* decreases. However, the monotonicity of *Q*, as a function of *δ* is much more complicated, as shown in Fig. 4(a) for the random immunization case. Generally speaking, *Q* is an increasing function of *δ* (Figs. 4(b)–4(d)). However, in Fig. 4(a), when the infection rate *β* is relatively small, say *β* = 0.2, the immunization efficiency is negatively correlated to the immunization strength, as highlighted by three blue lines. It is due to the dual effect of the increased immunization strength.^23^ Although increasing *δ* enlarges vaccination coverage, it also halts the spreading of an epidemic with a small infection rate across hub nodes and hence decreases propensity for vaccination. The relationship between *τ_c_* and *δ* is illustrated by dashed lines in each panel of Fig. 4. Almost all of these curves lie in the red region, showing that the immunization efficiency should be very high to control the disease. It is noticed that at the case *δ* = 1, the expression of *τ_c_* is reduced to be the same as the corresponding static immunization in Ref. 38. However, the spreading patterns between dynamic and static immunization are not the same, as many susceptible nodes are not vaccinated in Ω for the dynamic immunization.

## IV. CONCLUSION AND DISCUSSION

The study of the network theory enables us to analyze the role of each node or node set in the epidemic spreading and get novel insights into the transmission dynamics. As we know, the SIR-like epidemic network model can be analyzed by various approaches, such as the percolation theory,^43^ the branching process approximation,^16^ and the effective degree approaches.^32,44^ Recently, the heterogeneous mean-field approach poses a good tool to analyze complex disease dynamics due to its simple and deterministic formulation.^18,28^ In this manuscript, we formulated real-time immunization models with the discrete-time HMF approach, where susceptible nodes can get immunized by contact tracing from infected nodes. Considering the real situations of vaccine shortages such that the number of vaccines cannot cover the whole population, we propose a local immunization program that can only immunize a given node set Ω in the whole population, which can be defined as a geological region of a city or a social group of a population or other groups sharing some characteristics. The epidemic thresholds for different (local versus global) vaccination scenarios against infectious diseases are obtained from stability analysis, based on which the effectiveness of a vaccination program can be evaluated. The predicted thresholds are validated through numerical simulations. Our result suggests that the local immunization program can greatly improve the efficiency of static immunization, requiring a smaller amount of vaccines to effectively control disease spread. However, the efficacy of vaccination programs not only depends on immunization rate but also on the choice of individual group to immunize. Therefore, it remains pivotal to extend the approach in this manuscript to other local immunization strategies, with different targeted vaccination groups to get an optimal strategy for disease control. This may contribute toward the optimal strategy of vaccine allocation for emerging infectious diseases such as influenza A (H1N1).^45^

In the local immunization program with a SIR framework, we find that the immunization rate *δ* can greatly affect the epidemic threshold, which distinguishes from the prediction based on the SIS spreading mechanism where *δ* does not play a role in the threshold. This adds one more difference between the SIS and SIR network models, as revealed by Castellano and Pastor-Satorras^46^ that the threshold of generic epidemic models is vanishing for an SIS model, while it is finite for the SIR model on quenched scale-rich networks (i.e., *α* > 3).

In the present work, we only consider the same immunization rate *δ* for each node in the immunization set Ω. The same approach remains valid for a general case with multiple immunization sets with different *δ* values. Another interesting exploration may be the study of local immunization program in the interdependent networks^39^ or the community networks.^47^ These realistic issues suggest good topics for further research.

## APPENDIX: THE SVIS MODEL

In this appendix, we extend the SIS epidemic model with the consideration of dynamic immunization programs. For comparative purposes, the immunization and disease transmission parameters are set to be the same as those in the SVIR model. We denote $S_{k}^{\left(i\right)}(t),\;V_{k}^{\left(i\right)}(t)$, and $I_{k}^{\left(i\right)}(t)$ as the susceptible, vaccinated, infected densities among nodes inside (*i* = 1) and outside (*i* = 2) of the tracing group Ω with degree *k* at time step *t*, respectively. Then the following discrete-time SVIS model can be formulated for two different groups by using a similar approach as that in the main part:

$$\begin{array}{c} S_{k}^{\left(1\right)}(t+1)=S_{k}^{\left(1\right)}(t)\left\{1-E[w_{1}^{\left(1\right)}(s)]-E[w_{2}^{\left(1\right)}(s)]\right\}+\gamma I_{k}^{\left(1\right)}(t),\\ V_{k}^{\left(1\right)}(t+1)=V_{k}^{\left(1\right)}(t)+S_{k}^{\left(1\right)}(t)E[w_{1}^{\left(1\right)}(s)],\\ I_{k}^{\left(1\right)}(t+1)=(1-\gamma )I_{k}^{\left(1\right)}(t)+S_{k}^{\left(1\right)}(t)E[w_{2}^{\left(1\right)}(s)], \end{array}$$ (A1)

and

$$\begin{array}{c} S_{k}^{\left(2\right)}(t+1)=S_{k}^{\left(2\right)}(t)\left\{1-E[w_{2}^{\left(2\right)}(s)]\right\}+\gamma I_{k}^{\left(2\right)}(t),\\ I_{k}^{\left(2\right)}(t+1)=(1-\gamma )I_{k}^{\left(2\right)}(t)+S_{k}^{\left(2\right)}(t)E[w_{2}^{\left(2\right)}(s)]. \end{array}$$ (A2)

In this model, $w_{1}^{\left(1\right)}(s)=1-{\left(1-\delta \right)}^{s},\;w_{2}^{\left(1\right)}(s)={\left(1-\delta \right)}^{s}[1-{\left(1-\beta \right)}^{s}]$, and $w_{2}^{\left(2\right)}(s)=1-{\left(1-\beta \right)}^{s}$.

In order to obtain an approximate condition for epidemic outbreak, we set $S_{k}^{\left(i\right)}(t)=S_{k}^{\left(i\right)},\;I_{k}^{\left(i\right)}(t)=I_{k}^{\left(i\right)}$ for *i* = 1, 2 and $V_{k}^{\left(1\right)}(t)=V_{k}^{\left(1\right)}$ in Eqs. (A1) and (A2) where $S_{k}^{\left(i\right)},\;I_{k}^{\left(i\right)}$, and $V_{k}^{\left(1\right)}$ are constant values at the equilibrium state and furthermore, assume $I_{k}^{\left(1\right)}=0$ such that there is no infection in the set Ω due to immunization. Then the infectious proportion $I_{k}^{\left(2\right)}$ satisfies

$$\gamma I_{k}^{\left(2\right)}=[N_{k}^{\left(2\right)}-I_{k}^{\left(2\right)}][1-{\left(1-\beta \Theta \right)}^{k}],$$

where $N_{k}^{\left(2\right)}=S_{k}^{\left(2\right)}+I_{k}^{\left(2\right)}$ is a fixed value for each *k* and $\Theta =\frac{\sum_{k}kP(k)I_{k}^{\left(2\right)}}{\sum_{k}kP(k)}$. The positive solution for $I_{k}^{\left(2\right)}$ of the above algebraic equation exists if and only if $\tau :=\frac{\beta }{\gamma }>\frac{\sum_{k}kP(k)}{\sum_{k}N_{k}^{\left(2\right)}k^{2}P(k)}$. This inequality gives the epidemic threshold for the SVIS model, which is independent of the immunization strength *δ*. A similar result was claimed in the previous work.^23^
